# Factors associated with antiretroviral treatment adherence among people living with HIV in Guangdong Province, China: a cross sectional analysis

**DOI:** 10.1186/s12889-024-18774-6

**Published:** 2024-05-20

**Authors:** Jun Liu, Yao Yan, Yan Li, Kaihao Lin, Yingqian Xie, Zhimin Tan, Qicai Liu, Junbin Li, Lihua Wang, Yi Zhou, Gang Yao, Shanzi Huang, Chenglong Ye, Meixi Cen, Xiaowen Liao, Lu Xu, Chi Zhang, Yubin Yan, Lin Huang, Fang Yang, Yi Yang, Xiaobing Fu, Hongbo Jiang

**Affiliations:** 1 Department of HIV/AIDS Control and Prevention, Guangdong Provincial Center for Disease Control and Prevention, No. 160 Qunxian Road, Panyu District Guangzhou, Guangzhou, China; 2 Department of Epidemiology and Biostatistics, School of Public Health, Guangdong Pharmaceutical University, No. 283 Jianghai Road, Haizhu District, Guangzhou, 510310 China; 3 Affiliated Hospital of Guangdong Medical University, Zhanjiang, China; 4 Guangzhou Eighth People’s Hospital, Guangzhou Medical University, Guangzhou, China; 5 Jiangmen Center for Disease Control and Prevention, Jiangmen, China; 6 Zhuhai Center for Disease Control and Prevention, Zhuhai, China; 7 Yangjiang Center for Disease Control and Prevention, Yangjiang, China; 8 Yunfu Center for Disease Control and Prevention, Yunfu, China; 9 Shantou Center for Disease Control and Prevention, Shantou, China; 10 Huizhou Center for Disease Control and Prevention, Huizhou, China; 11 Institute for Global Health, University College London, London, NW3 2PF United Kingdom

**Keywords:** People living with HIV, Antiretroviral treatment, Adherence, Associated factors, Psychosocial effect, Structural equation modeling

## Abstract

**Background:**

Understanding factors associated with antiretroviral treatment (ART) adherence is crucial for ART success among people living with HIV (PLHIV) in the “test and treat” era. Multiple psychosocial factors tend to coexist and have a syndemic effect on ART adherence. We aimed to explore factors associated with ART adherence and the syndemic effect of multiple psychosocial factors on ART adherence among PLHIV newly starting ART in Guangdong Province, China.

**Methods:**

Newly diagnosed PLHIV from six cities in Guangdong Province were recruited between May 2018 and June 2019, and then followed up from May 2019 to August 2020. Baseline and follow-up data were collected from a questionnaire and the national HIV surveillance system, the follow-up data of which were analyzed in this study. A Center for Adherence Support Evaluation (CASE) index > 10 points was defined as optimal ART adherence, which was measured via participants’ self-reported adherence during follow-up survey. Multivariable logistic regression was used to identify factors associated with ART adherence. Exploratory factor analysis (EFA) and multi-order latent variable structural equation modeling (SEM) were performed to explore the syndemic effect of multiple psychosocial factors on ART adherence.

**Results:**

A total of 734 (68.53%) follow-up participants were finally included in this study among the 1071 baseline participants, of whom 91.28% (670/734) had self-reported optimal ART adherence. Unemployment (aOR = 1.75, 95%CI: 1.01–3.02), no medication reminder (aOR = 2.28, 95%CI: 1.09–4.74), low medication self-efficacy (aOR = 2.28, 95%CI: 1.27–4.10), low social cohesion (aOR = 1.82, 95%CI: 1.03–3.19), no social participation (aOR = 5.65, 95%CI: 1.71–18.63), and ART side effects (aOR = 0.46, 95%CI: 0.26–0.81) were barriers to optimal ART adherence. The EFA and second-order latent variable SEM showed a linear relationship (standardized coefficient = 0.43, *P* < 0.001) between ART adherence and the latent psychosocial (syndemic) factor, which consisted of the three latent factors of medication beliefs and self-efficacy (standardized coefficient = 0.65, *P* < 0.001), supportive environment (standardized coefficient = 0.50, *P* < 0.001), and negative emotions (standardized coefficient=-0.38, *P* < 0.01). The latent factors of medication beliefs and self-efficacy, supportive environment, and negative emotions explained 42.3%, 25.3%, and 14.1% of the variance in the latent psychosocial factor, respectively.

**Conclusions:**

About nine out of ten PLHIV on ART in Guangdong Province self-reported optimal ART adherence. However, more efforts should be made to address barriers to optimal ART adherence.

**Supplementary Information:**

The online version contains supplementary material available at 10.1186/s12889-024-18774-6.

## Background

In the era of “test and treat”, optimal adherence to antiretroviral treatment (ART) plays a crucial role as more people living with HIV (PLHIV) start ART. However, suboptimal ART adherence is prevalent and detrimental to effective HIV viral suppression, increasing the risk of transmission, accumulation of resistance mutations, disease progression, and death [1]. A systematic review and meta-analysis showed that by May 2015, only 77.6% (95%CI: 71.6–83.1) of PLHIV in China had adequate ART adherence, defined as the proportion of people reporting ≥ 90% adherence to ART [2]. Another study showed that by October 2016, the pooled proportion of ≥ 95% adherence to ART was 81.1% (95%CI: 75.1–88.0) at one week and decreased over time among PLHIV in China [3]. A recent study showed that the proportion of good ART adherence was 53.4% among men who have sex with men (MSM) with new drug abuse in Jinan, China [4]. Therefore, more efforts should be made to improve ART adherence, which is critical to achieve the 95-95-95 targets by 2030.

Previous studies showed that gender, transmission route, education level, substance use, medication reminders, side effects, ART duration, beliefs about ART, self-efficacy, social support, depression, HIV-related stigma, etc. were associated with ART adherence among PLHIV in China [5–10]. Another study showed that having higher perceived social capital was a significant predictor of better HIV ART adherence among PLHIV in North America [11]. Among the factors associated with ART adherence, psychosocial factors, including beliefs about ART, self-efficacy, social support, social capital, depression, HIV-related stigma, etc., could be targeted as modifiable factors for interventions to improve ART adherence and are receiving increasing attention. However, psychosocial factors are more likely to coexist and may interact with each other to influence ART adherence, which could be addressed by applying syndemic theory [12–14].

Syndemic theory is concerned with the idea that psychosocial problems tend to co-occur and interact with each other, amplifying their impact on health outcomes among PLHIV and those at risk of HIV infection [12, 13]. Previous studies showed that more syndemic factors (including depression, HIV-related stigma, substance use, violence, etc.) were associated with a higher risk of suboptimal ART adherence [14–18]. However, the additive associations of different syndemic factors with ART adherence may not actually explain the effects of interactions between the syndemic factors on ART adherence and may provide limited guidance for intervention efforts [19–21]. Therefore, alternative methods should be used to examine the syndemic burden formed by the interaction of psychosocial factors on ART adherence. Structural equation modeling (SEM) has been used to construct a latent syndemic component to help understand the interactions between different psychosocial factors and their effects on suicide attempts [20] and the number of condomless anal sex partners [21] among sexual minority men in previous studies, which may be an optimal approach to explore the syndemic effect of psychosocial factors on ART adherence.

China has implemented the National Free ART Program since 2003, providing free ART to PLHIV who meet the national treatment guidelines, which has been expended to all PLHIV in the era of “test and treat” since 2016 [22, 23]. Guangdong Province ranked fifth in China in terms of the largest number of PLHIV by the end of 2014 [24], and a cumulative 81,641 cases of HIV infection had been reported in Guangdong, of which 33,993 had progressed to AIDS and 21,006 had died by the end of 2017 [25]. A total of 53,639 PLWH in Guangdong had received free ART, with 44,190 still receiving treatment in Guangdong by the end of 2017 [25]. However, ART adherence has not been adequately studied since the implementation of the “test and treat” policy in Guangdong Province. Therefore, we conducted this study to understand the prevalence and correlates of suboptimal ART adherence among PLHIV newly starting ART in the era of “test and treat” in Guangdong Province, and to further explore the syndemic effect of multiple psychosocial factors on ART adherence.

## Methods

### Settings and participants

A prospective cohort study was conducted in six cities in Guangdong Province, which were selected on the basis of the geographical location and economic status: Shantou City in eastern Guangdong Province, Yangjiang City in western Guangdong Province, Yunfu City in northern Guangdong Province, Zhuhai City, Jiangmen City, and Huizhou City in the Pearl River Delta (the most developed area in Guangdong Province). At baseline, participants were recruited using consecutive sampling with the following inclusion criteria: aged ≥ 18 years, currently living in Guangdong Province, newly diagnosed with HIV infection and reported to the national HIV surveillance system between May 2018 and June 2019, and able to understand the study objectives and procedures, and able to provide written informed consent. After signing the written informed consent, they were invited to complete a questionnaire, when they presented to the trained staff at six Centers for Disease Prevention and Control (CDCs) to report their confirmed HIV-positive result, as described previously [24]. Routine follow-up of PLHIV was conducted according to the guidelines of the China comprehensive AIDS response program, as described previously [26]. One year after the baseline survey (from May 2019 to August 2020), the trained medical staff at the local CDCs or HIV-designated hospitals made an appointment with the participants to complete a follow-up questionnaire. The study was conducted in accordance with the Declaration of Helsinki (as revised in 2013). This study was approved by the ethics committee of Guangdong Pharmaceutical University. No incentives were offered to the participants. They were asked to sign a written informed consent document before participating in the study.

### Measurements

Data were collected at baseline and follow-up using a questionnaire and the national HIV surveillance system. Data from the questionnaire and the national HIV surveillance system were matched using unique identification from the infectious disease notification card. At baseline, the questionnaire collected data on HIV-related symptoms within one year of diagnosis, health insurance, income, social capital, social support, HIV stigma, depression, etc. Data on socio-demographic information (age, sex, marital status, etc.) and other HIV-related information (transmission route, sample source, CD4 cell count, etc.) were obtained from the national HIV surveillance system. At follow-up, data were collected on ART, adherence to ART, beliefs about medication, ART adherence self-efficacy, adverse effects of ART, medication reminders, reasons for skipping medication, and never skipping medication, except for those were collected at baseline. Notably, data on adherence were collected only at follow-up from May 2019 to August 2020, and therefore only data at follow-up were analyzed in the current study.

### ART adherence

The Center for Adherence Support Evaluation (CASE) Adherence Index was used to measure ART adherence, which consists of three self-reported items: frequency of taking ART medication on time (not more than two hours before or two hours after the time your doctor told you to take it; responses were never, rarely, most of the time, or always), average number of days per week with at least one missed dose (responses were: every day, 4–6 days a week, 2–3 days a week, once a week, less than once a week, or never), and last time at least one dose of ART medication was missed (responses were: within the last week, 1–2 weeks ago, 3–4 weeks ago, between one and three months ago, more than three months ago, or never) [27]. Responses to the three items were coded as 1 to 4 scores for the first item and 1 to 6 scores for the second and third items, which were summed to produce a composite score (Cronbach’s α = 0.69), ranging from 3 to 16, with higher scores indicating better adherence. Scores > 10 indicated optimal adherence, and conversely, suboptimal adherence.

### Beliefs about medication

The beliefs about medicines questionnaire specific (BMQ-S) was used to measure beliefs about medication, which includes two five-item scales, the specific-necessity (BMQ-SN) and specific-concerns (BMQ-SC) scales [28]. Respondents were asked to indicate their level of agreement with each item on a five-point Likert scale, ranging from 1 = strongly disagree to 5 = strongly agree. Items in each subscale were rated on a five-point Likert scale ranging from 1 (strongly disagree) to 5 (strongly agree), resulting in total scores ranging from 5 to 25 (Cronbach’s α = 0.78). Higher scores indicate stronger beliefs. A necessity-concerns differential is calculated as the difference between the BMQ-SN and the BMQ-SC scales, with a possible range of -20 to + 20. If the difference is positive, this indicates a predominance of perceived necessity to take medication and, conversely, a predominance of concern about taking medication.

### ART adherence self-efficacy

A 12-item HIV Treatment Adherence Self-Efficacy Scale (HIV-ASES) was used to measure efficacy of adherence to ART [29]. Respondents were asked to rate their confidence in performing important treatment-related behaviors related to adherence to treatment plans on an eleven-point Likert scale, ranging from 0 (cannot do it at all) to 10 (certainly can do it) (Cronbach’s α = 0.98). Higher scores indicate higher adherence self-efficacy. The median score was used as a cut-off value to classify low and high levels of adherence self-efficacy.

### Social support

The Social Support Rating Scale (SSRS) was used to measure social support [30]. Items were rated on a four-point scale ranging from 1 to 4. Responses to the ten items were summed to obtain a composite score (Cronbach’s α = 0.73). The median score was used as a cut-off value to classify low and high levels of social support.

### Social capital

Social capital was measured in terms of social trust, social participation, social cohesion, and collective engagement [31–33], which were described in detail in our previous study [24]. A “yes” response to the single-item questions on social trust and collective engagement was classified as social trust and collective engagement. An answer to the single-item question on social participation with one or more options was classified as “yes” for social participation. Social cohesion was assessed by four items, three of which used a 4-point scale ranging from 1 (strongly disagree) to 4 (strongly agree) and one of which used a 5-point scale ranging from 1 (never) to 5 (always) (Cronbach’s α = 0.68). The median score was used as a cut-off value to classify low and high levels of social cohesion.

#### Depression

Depression was measured using the 20-item Center for Epidemiologic Studies-Depression (CES-D) scale [34]. Items were scored on a 4-point scale from 0 to 3, resulting in total scores ranging from 0 to 60 (Cronbach’s α = 0.90). Scores ≥ 28 indicated depression.

### HIV-related stigma

The revised 10-item Berger’s HIV Stigma Scale was used to measure perceived HIV-related stigma [35, 36]. Items were rated on a four-point Likert scale, ranging from 1 (strongly disagree) to 4 (strongly agree), resulting in total scores ranging from 10 to 40 (Cronbach’s α = 0.87). Higher scores indicate higher levels of perceived HIV-related stigma. The median score was used as a cut-off value to classify low and high levels of perceived HIV-related stigma.

### Statistical analysis

Mean and standard deviation were used to describe normally distributed variables, and median and IQR were used for non-normally distributed variables. *χ*^2^ tests were used to compare the differences in suboptimal ART adherence according to socio-demographic, HIV-related, and psychosocial variables (beliefs about medication, ART adherence self-efficacy, social support, social capital, depression and HIV-related stigma). Statistically significant variables from the *χ*^2^ tests were included in the multivariable logistic regression models to calculate the adjusted odds ratios (a*OR*s) with 95% confidence intervals (*CI*s). In order to further explore the syndemic effect of multiple psychosocial factors on ART adherence, Pearson’s correlation analysis was used to examine the correlations between the psychosocial factors and the three components of the CASE Adherence Index. An exploratory factor analysis (EFA) was performed to identify the main latent constructs underlying the multiple psychosocial factors. Varimax orthogonal rotation was applied and factors were extracted when eigenvalues > 1 [37]. First-order and multi-order latent variable SEMs were used to explore the syndemic effect of multiple psychosocial factors on ART adherence. The absolute, parsimony and comparative fit indices were used to assess the goodness-of-fit of the SEM. Data analyses were performed using SAS version 9.4 (SAS Institute Inc., Cary, NC, USA). All hypothesis tests were 2-tailed with α = 0.05.

## Results

### Sociodemographic characteristics

A total of 1071 newly reported PLHIV were recruited in the baseline survey from May 2018 to June 2019. Of the 792 (73.95%) who completed the follow-up survey, 734 (92.68%) on ART were included in this study. The median age of the participants was 44.08 (IQR: 31.06–56.07) years old. Most of them were male (78.47%), had health insurance (84.33%), had a middle school education or less (65.12%), were transmitted through heterosexual contact (65.12%), and were diagnosed in medical institutions (65.12%, Table 1).

**Table 1:** Association between demographic, HIV-related characteristics and antiretroviral treatment adherence among people living with HIV in guangdong province, China

Characteristic	*N*(%)	ART adherence^†^		χ^2^	*P*
		Optimal (%)	Suboptimal (%)		
Gender				1.808	0.179
Male	576(78.47)	530(92.01)	46(7.99)		
Female	158(21.53)	140(88.61)	18(11.39)		
Age (years)				1.653	0.199
< 44	366(49.86)	339(92.62)	27(7.38)		
≥ 44	368(50.14)	331(89.95)	37(10.05)		
Registered residence				0.383	0.536
Guangdong Province	540(73.57)	495(91.67)	45(8.33)		
Other provinces	194(26.43)	175(90.21)	19(9.79)		
Education				2.199	0.333
Junior high school or below	478(65.12)	431(90.17)	47(9.83)		
Senior high school or equivalent	142(19.35)	132(92.96)	10(7.04)		
Tertiary education or above	114(15.53)	107(93.86)	7(6.14)		
Marital status				2.353	0.125
Unmarried/divorced/widowed	424(57.49)	391(92.65)	31(7.35)		
Married	312(42.51)	279(89.42)	33(10.58)		
Monthly income (Yuan)				0.820	0.664
0	145(19.75)	132(91.03)	13(8.97)		
1 ∼ < 3000	231(31.47)	214(92.64)	17(7.36)		
≥3000	358(48.77)	324(90.50)	34(9.50)		
Employment				4.062	**0.044**
No	292(39.78)	259(88.70)	33(11.30)		
Yes	442(60.22)	411(92.99)	31(7.01)		
Medical insurance				0.532	0.466
No/unknown	115(15.67)	107(93.04)	8(6.96)		
Yes	619(84.33)	563(90.95)	56(9.05)		
Transmission route				3.398^§^	0.183
Heterosexual contact	478(65.12)	430(89.96)	48(10.04)		
Men who have sex with men	233(31.74)	219(93.99)	14(6.01)		
Others	23(3.13)	21(91.30)	2(8.70)		
Sample source^¶^				1.118^§^	0.622
Medical institutions	478(65.12)	435(91.00)	43(9.00)		
VCT clinics	213(29.02)	194(91.08)	19(8.92)		
Other	43(5.86)	41(95.35)	2(4.65)		
Medication side effects and their impact				15.590	**< 0.001**
No side effects or mild side effects without impact on daily life	547(74.52)	510(93.24)	37(6.76)		
Side effects with mild impact on daily life	165(22.48)	143(86.67)	22(13.33)		
Side effects with major impact on daily life or serious side effects	22(3.00)	17(77.27)	5(22.73)		
Taking medicine with reminder				5.564	**0.018**
No	75(10.22)	63(84.00)	12(16.00)		
Yes	659(89.78)	607(92.11)	52(7.89)		

^†^ ART: antiretroviral treatment

^§^ Likelihood-ratio chi-square

^¶^ Medical institutions: testing before surgery, blood transfusion and donation, invasive examination, premarital and prenatal examination, sexually transmitted disease clinics and so on. VCT clinics: testing in voluntary counseling and testing clinics

### Psychosocial factors

The median BMQ-necessity score, BMQ-concern score, and BMQ-necessity-concern score were 19 (IQR: 17–21), 15 (IQR: 13–18), and 2 (IQR: 0–6), respectively. The proportion of participants whose BMQ-necessity score was higher than their BMQ-concern score was 69.62%. The median ART adherence self-efficacy score, social support score, social cohesion score and HIV-related stigma score were 114 (IQR: 97 ∼ 120), 33 (IQR: 29 ∼ 38), 9 (IQR: 8 ∼ 10), and 27 (IQR: 24 ∼ 30), respectively. The proportions of social participation, collective engagement, social trust, and high social cohesion were 18.80%, 13.90%, 41.14%, and 70.71%, respectively. More than half of the participants had high self-efficacy for ART adherence (50.68%), high social support (55.31%), and high HIV-related stigma (56.40%). There were 89 (12.13%) participants with a CES-D score ≥ 28 who were classified as depression (Table 2).

**Table 2:** Association between psychosocial factors and antiretroviral treatment adherence among people living with HIV in guangdong province, China

Characteristic	*N*(%)	ART adherence^†^		χ^2^	*P*
		Optimal (%)	Suboptimal (%)		
The beliefs about medicines				4.620	**0.032**
Necessity > Concerns	511(69.62)	474(92.76)	37(7.24)		
Necessity≤Concerns	223(30.38)	196(87.89)	27(12.11)		
Adherence self-efficacy				10.591	**0.001**
Low	362(49.32)	318(87.85)	44(12.15)		
High	372(50.68)	352(94.62)	20(5.38)		
HIV-related stigma				1.060	0.303
Low	320(43.60)	296(92.50)	24(7.50)		
High	414(56.40)	374(90.34)	40(9.66)		
Social cohesion				10.466	**0.001**
Low	215(29.29)	185(86.05)	30(13.95)		
High	519(70.71)	485(93.45)	34(6.55)		
Social participation				9.148	**0.003**
No	596(81.20)	535(89.77)	61(10.23)		
Yes	138(18.80)	135(97.83)	3(2.17)		
Collective engagement				0.513	0.474
No	632(86.10)	575(90.98)	57(9.02)		
Yes	102(13.90)	95(93.14)	7(6.86)		
Social trust				0.197	0.658
No	432(58.86)	396(91.67)	36(8.33)		
Yes	302(41.14)	274(90.73)	28(9.27)		
Depression				6.255	**0.012**
No	645(87.87)	595(92.25)	50(7.75)		
Yes	89(12.13)	75(84.27)	14(15.73)		
Social support					
Low	328(44.69)	297(90.55)	31(9.45)	0.399	0.528
High	406(55.31)	373(91.87)	33(8.13)		

^†^ ART: antiretroviral treatment

### ART adherence

**Fig. 1:**
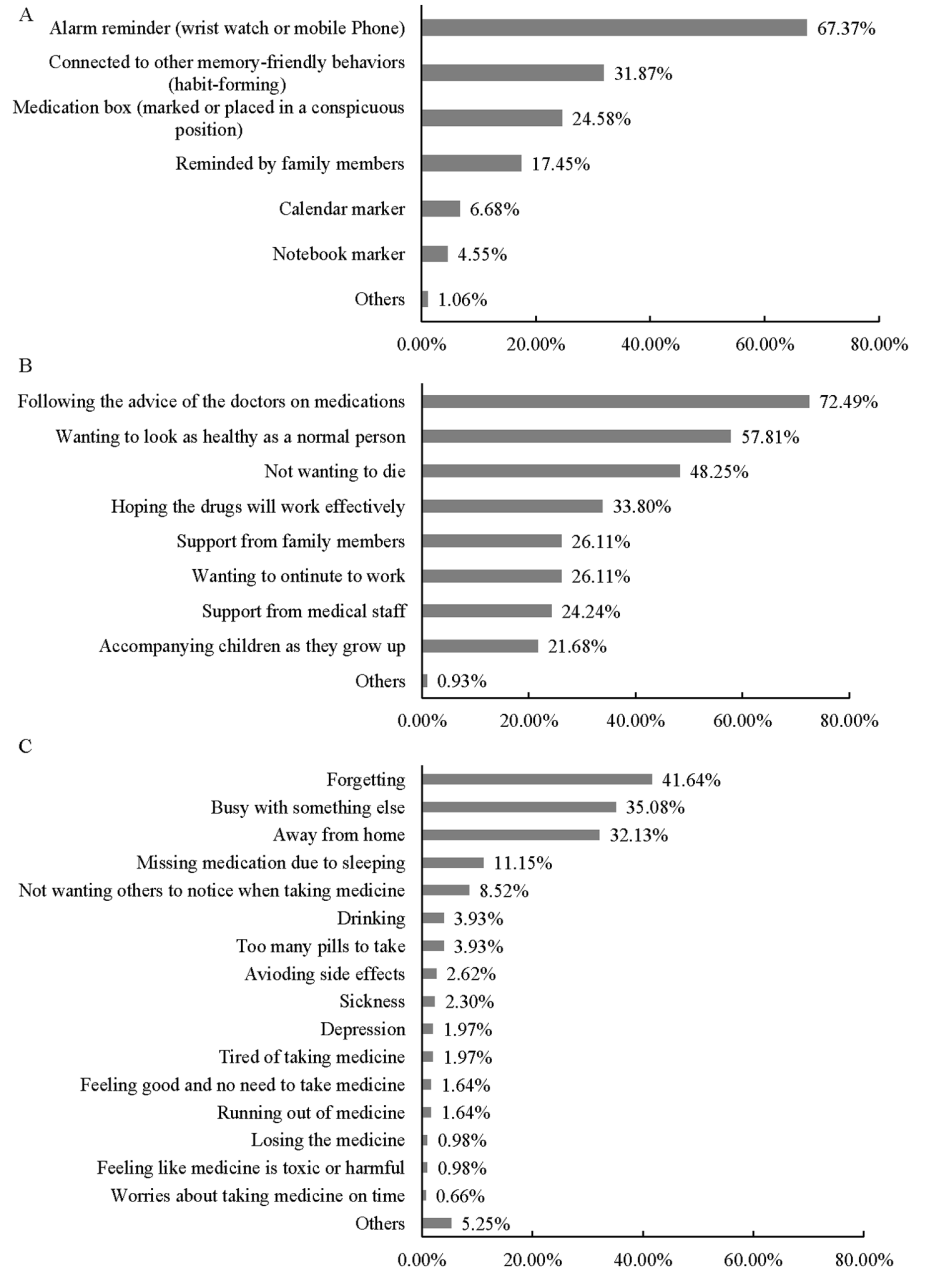
The medication reminder methods and reasons for skipping and never skipping medication among people living with HIV in guangdong province, China **A**: Medication reminder methods; **B**: Reasons for skipping medication; **C**: Reasons for never skipping medication

Around three quarters (74.52%) of participants reported no medication side effects (49.59%) and reported mild side effects that did not affect their daily life (24.93%) in the last two weeks. The majority (89.78%) of participants had reminders to help them take their medication on time, with the most common reminder being a watch or phone alarm (Fig. 1A). Of the participants, 670 (91.28%) with a CASE score > 10 were defined as having optimal adherence. Among the 429 (58.45%) participants who never skipped their ART medication and took it on time, the top three reasons for never skipping their medication were following the advice of the doctors (72.49%), wanting to look as healthy as normal (57.81%), and not wanting to die (48.25%, Fig. 1B). Among the remaining 305 (41.55%) participants who skipped their ART medication or could not take it on time, the top three reasons for skipping their medication were forgetting (41.64%), being busy with something else (35.08%), and being away from home (32.13%, Fig. 1C).

### Factors associated with ART adherence

Univariate analysis showed that employment, side effects and impact on daily life, medication reminders, beliefs about medicine, ART adherence self-efficacy, social cohesion, social participation and depression were associated with ART adherence (*P* < 0.05, Tables 1 and 2). After adjusting for statistically significant variables in univariate analyses, multivariable logistic regression showed that participants who were unemployed (aOR = 1.75, 95% CI: 1.01 to 3.02), had no medication reminders (aOR = 2.28, 95% CI: 1.09 to 4.74), low ART adherence self-efficacy (aOR = 2.28, 95% CI: 1.27 to 4.10), low social cohesion (aOR = 1.82, 95% CI: 1.03 to 3.19), and no social participation (aOR = 5.65, 95% CI: 1.71 to 18.63) were associated with a higher risk of suboptimal adherence. Individuals who reported no side effects or no impact on daily life (aOR = 0.46, 95% CI: 0.26 to 0.81) were less likely to have suboptimal adherence than those who reported side effects which affected daily life (Table 3).

**Table 3:** Factors associated with suboptimal antiretroviral treatment adherence among people living with HIV in guangdong province, China

Characteristic	aOR(95%CI) ^†^	*P*
Employment		
Yes	1.00	
No	1.75(1.01 ∼ 3.02)	**0.045**
Medication side effects and their impact		
Side effects with impact on daily life	1.00	
No side effects or mild side effects without impact on daily life	0.46(0.26 ∼ 0.81)	**0.007**
Taking medicine with reminder		
Yes	1.00	
No	2.28(1.09 ∼ 4.74)	**0.028**
The beliefs about medicines		
Necessity ≤ Concerns	1.00	
Necessity > Concerns	0.77(0.43 ∼ 1.38)	0.381
Adherence self-efficacy		
Higher	1.00	
Lower	2.28(1.27 ∼ 4.10)	**0.006**
Social cohesion		
Higher	1.00	
Lower	1.82(1.03 ∼ 3.19)	**0.039**
Social participation		
Yes	1.00	
No	5.65(1.71 ∼ 18.63)	**0.005**
Depression		
Yes	1.00	
No	0.61(0.31 ∼ 1.22)	0.163

^†^ aOR (95%CI): adjusted odds ratio (95% confidence interval)

^§^ Adjusted for employment, medication side effects and their impact, taking medicine with reminder, the beliefs about medicines, adherence self-efficacy, social cohesion, social participation and depression

### Syndemic effect of psychosocial factors on ART adherence using SEM and EFA

**Fig. 2:**
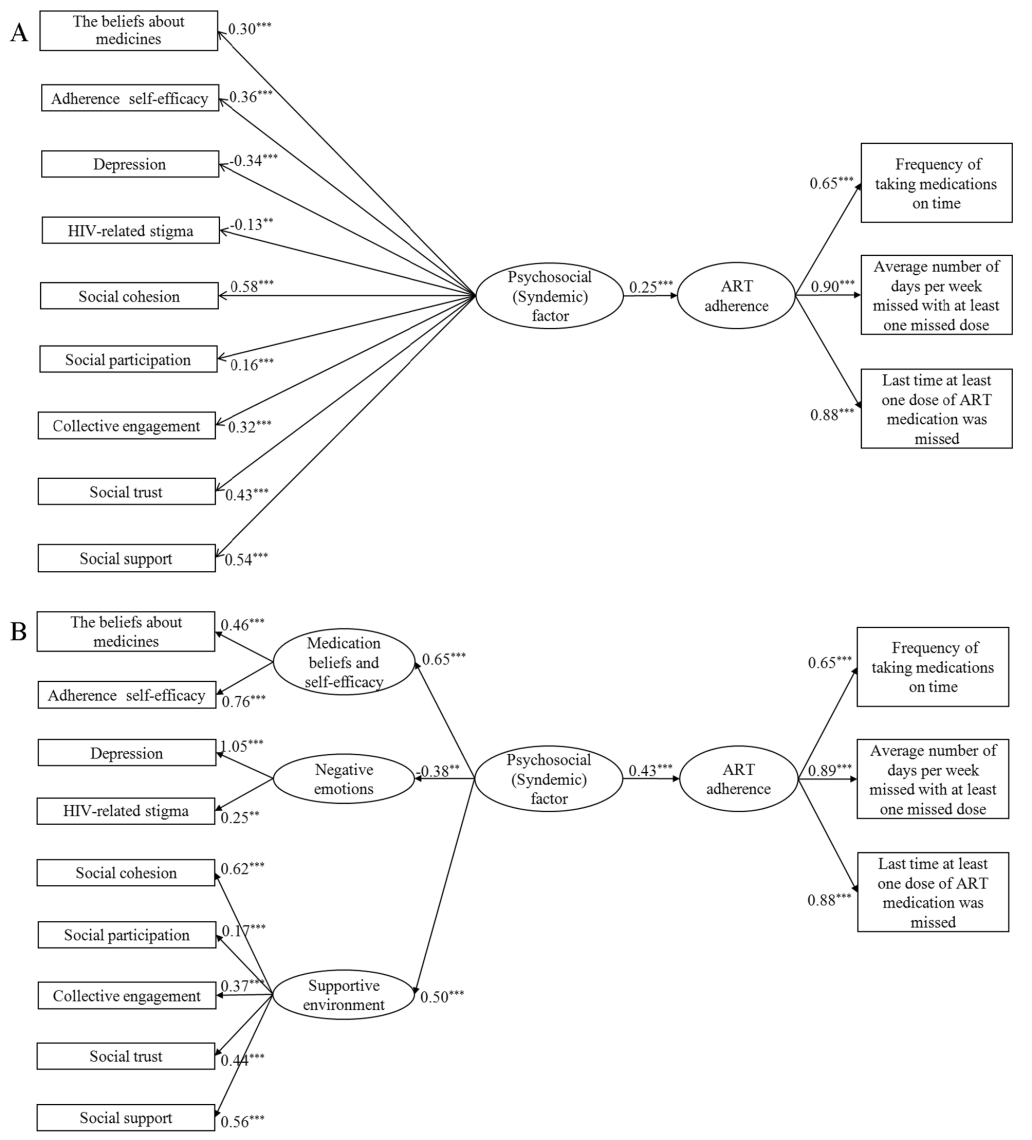
The latent symdemic effect of multiple psychosocial factors on antiretroviral treatment (ART) adherence using structural equation models **A**: First-order latent variable structural equation model; **B**: Second-order latent variable structural equation model Note: All values presented are standardized coefficient. ^**^P < 0.01; ^***^P < 0.001

As shown in Table 4, not all psychosocial factors and the three components of the CASE Adherence Index were related to each other. A first-order SEM was then fitted to the data, with all 9 psychosocial factors loading on the latent psychosocial (syndemic) factor (Fig. 2A). However, not all the goodness-of-fit indices met the recommended criteria (*χ*^2^/*df* = 5.59 > 3, root mean square residual, RMR = 0.07 > 0.05, Table S1). In addition, the latent psychosocial factor was associated with ART adherence (standardized coefficient = 0.25, *P* < 0.001), and could explain only 6.2% of the variance in ART adherence (R^2^ = 0.062, Table S2).

**Table 4:** The correlation matrix among psychosocial factors and antiretroviral treatment adherence people living with HIV in guangdong province, China

No.	Construct	1		2		3		4		5		6		7		8		9		10		11	12		
1	The beliefs about medicines		1.000																						
2	Adherence self-efficacy		0.347^***^		1.000																				
3	HIV-related stigma		-0.053		-0.034		1.000																		
4	Social cohesion		0.119^**^		0.166^***^		0.013		1.000																
5	Social participation		-0.060		-0.064		-0.079^*^		0.078^*^		1.000														
6	Collective engagement		0.036		0.030		-0.009		0.202^***^		0.149^***^		1.000												
7	Social trust		0.197^***^		0.121^***^		0.010		0.270^***^		-0.013		0.272^***^		1.000										
8	Social support		0.083^*^		0.145^***^		-0.114^**^		0.356^***^		0.152^***^		0.192^***^		0.212^***^		1.000								
9	Depression		-0.093^*^		-0.164^***^		0.267^***^		-0.191^***^		-0.143^***^		0.048		-0.077^*^		-0.193^***^		1.000						
10	The CASE Adherence Index questions 1^†^		0.127^***^		0.230^***^		-0.056		0.117^**^		0.079^*^		0.023		-0.014		0.055		-0.122^***^		1.000				
11	The CASE Adherence Index questions 2^§^		0.089^*^		0.209^***^		-0.026		0.089^*^		0.059		0.049		0.048		0.038		-0.157^***^		0.579^***^	1.000			
12	The CASE Adherence Index questions 3^¶^		0.102^**^		0.228^***^		-0.033		0.106^**^		0.061		0.049		0.029		0.075^*^		-0.146^***^		0.565^***^	0.788^***^	1.000		

† Frequency of taking medications on time

§ Average number of days per week missed with at least one missed dose

¶ Last time at least one dose of ART medication was missed

*P<0.05，**P<0.01，***P<0.001

Therefore, the EFA was conducted to identify the main latent constructs underlying the multiple psychosocial factors. The EFA showed that the nine psychosocial factors were loaded on three latent factors (Table 5), including a latent factor of medication beliefs and self-efficacy, a factor of negative emotions (HIV-related stigma and depression), and a factor of supportive environment (social trust, social participation, social cohesion, collective engagement and social support).

**Table 5:** Rotation factor loading matrix from exploratory factor analysis of psychosocial factors

Psychosocial factor	Factor 1	Factor 2	Factor 3
	Medication beliefs and self-efficacy factor	Negative emotions factor	Supportive environment factor
The beliefs about medicines	**0.730**	-0.019	0.169
Adherence self-efficacy	**0.664**	-0.202	0.056
HIV-related stigma	0.012	**0.692**	0.097
Social cohesion	0.165	-0.153	**0.628**
Social participation	-0.442	-0.381	**0.305**
Collective engagement	-0.165	0.145	**0.683**
Social trust	0.244	0.147	**0.642**
Social support	0.003	-0.387	**0.590**
Depression	-0.208	**0.712**	-0.078

A second-order SEM was further fitted to the data with the three latent factors loaded by 9 psychosocial factors, further loading on the latent psychosocial (syndemic) factor (Fig. 2B). The latent psychosocial (syndemic) factor consisted of the three latent factors of medication beliefs and self-efficacy (standardized coefficient = 0.65, *P* < 0.001), supportive environment (standardized coefficient = 0.50, *P* < 0.001), and negative emotions (standardized coefficient=-0.38, *P* < 0.01). All the goodness-of-fit indices met the recommended criteria (Table S3). The latent psychosocial factor was associated with ART adherence (standardized coefficient = 0.43, *P* < 0.001), and could explain 18.6% of the variance in ART adherence (R^2^ = 0.186). In addition, the latent factors of medication beliefs and self-efficacy, supportive environment and negative emotions explained 42.3%, 25.3%, and 14.1% of the variance in the latent psychosocial factor, respectively (Table S4).

## Discussion

Our results showed that 91.28% of PLHIV on ART in Guangdong Province self-reported optimal adherence to ART. However, side effects of ART affecting daily life, unemployment, HIV treatment adherence self-efficacy, social cohesion and participant were independently associated with adherence to ART. A latent psychosocial variable was constructed from different dimensions of psychosocial factors and found to be associated with adherence to ART, which may help to better understand the variation of psychosocial effects on adherence to ART and tailor targeted interventions to improve adherence to ART.

The proportion of optimal adherence to ART, as measured by the CASE Adherence Index, in Guangdong Province was higher than that of women living with HIV in rural Eswatini (44%, 73/166) [38], people living with HIV in a UK sexual health and HIV clinic (88%, 190/227) [39], women living with HIV in South Africa (64.3%, 391/608) [40], HIV-infected MSM with new drug abuse in Jinan City (53.4%, 157/294) [4], but similar to that of PLHIV in Nanjing City (91.3%, 252/276) [41] and Hunan Province (94.6%, 175/185)) [42]. Two previous systematic reviews and meta-analyses showed that among PLHIV in China, the pooled proportion of ≥ 90% adherence to ART was 77.6% by May 2015 [2] and the pooled proportion of ≥ 95% adherence to ART was 81.1% by October 2016 [3]. Lower levels of ART adherence have also been observed among PLHIV in Yunnan Province and the Guangxi Zhuang Autonomous Region [43–45]. The different levels of optimal ART adherence may be attributed to differences in study settings, populations [4, 38–42], and definitions of optimal ART adherence [2, 3, 43–45], and so on. Although about nine out of ten participants self-reported optimal ART adherence, 41.55% of participants had missed medication. Forgetting to take medication and having a busy schedule were the most commonly reported reasons for missing medication, which may be due to lack of medication reminders. In addition, lack of medication reminders was found to be a risk factor for suboptimal ART adherence in our study, which was also supported by previous studies [46, 47]. Therefore, appropriate reminders play an important role in ensuring that PLHIV take their medications on time. The most common reminder in our study is a watch or phone alarm, which has also been reported to facilitate optimal ART adherence in previous studies [48, 49]. In addition, nearly one in three PLHIV missed their medication when they were away from home. In a previous study, people with HIV preferred the long-acting HIV regimens to facilitate travel [50]. Our findings underscore the need to motivate health care providers to improve access to antiretroviral drugs and to find appropriate treatment options that fit into the lives of PLHIV [50, 51].

Side effects of ART that affected daily life were found to be a barrier to optimal adherence. A recent study found that the experience of ART side effects was negatively correlated with ART satisfaction, which was positively correlated with optimal ART adherence [51]. Side effects of ART were also reported to be a predictor of suboptimal ART adherence among PLHIV in Shenzhen [8]. This finding highlighted the need for patient-centered care to reduce the side effects of ART and improve ART adherence.

Unemployment is a risk factor for suboptimal adherence to ART, which is in line with a previous systematic review and meta-analysis showing that employed PLHIV, particularly those in low- and high-income countries, were more likely to adhere to ART than those who were unemployed [52]. China has launched the National Free ART Program since 2003, providing free ART to PLHIV who meet the national treatment guidelines, which has been expanded to all PLHIV in the era of test and treat since 2016 [22, 23]. However, other expenses, rather than the cost of ART, remained a significant financial burden for people on lifelong ART [5, 53–55]. Financial constraints may be a proxy for unemployment, which is associated with suboptimal adherence to ART. Employment may be associated with increased social support, better time management, improved psychosocial and material well-being, and improved access to health services, contributing to optimal ART adherence [52, 56].

HIV treatment adherence self-efficacy is the confidence in performing important treatment-related behaviors related to adherence to treatment plans, which has been reported as a predictor of ART adherence [57]. Self-efficacy is derived from social cognitive theory, which posits that effective self-regulation and behavior change require self-confidence and self-motivation [58], although increased awareness and knowledge of health risks are important components of change. In addition, a lack of self-efficacy can be the cause of further inconsistencies between knowledge and action. Therefore, efforts might be made to improve HIV treatment adherence self-efficacy to ensure better treatment management, particularly with regard to medication schedules and taking all doses on time [59].

Social capital is conceptualized as the collective resources generated through social connections that individuals or groups can access. High levels of social capital may be associated with increased supportive social norms, increased information sharing and reduced HIV/AIDS stigma [32], contributing to optimal ART adherence [60]. An ethnographic study found that fulfilling social responsibilities, and thus maintaining social capital in key relationships, contributed to successful adherence in sub-Saharan Africa [61]. However, few studies have focused on the effect of social capital on ART adherence in China. The positive effects of social cohesion and social participation on ART adherence emphasize the importance of social networks and the collective ability of PLHIV to overcome the challenges they face [11].

The main strength of our study is the application of syndemic theory to understand the impact of psychosocial factors on ART adherence. Previous studies showed that more syndemic psychosocial factors were associated with lower ART adherence among PLHIV [14, 16, 62]. When examining the relationship between the number of psychosocial factors and ART adherence, it was assumed that the effects of each psychosocial factor were equal. However, the syndemic effect of the interaction between multiple psychosocial factors is inconsistent with the simple additive effect of a single psychosocial factor [19–21]. The positive dose-response relationship between the number of psychosocial factors and adherence to ART may not actually explain the relationship between the interactions among the psychosocial factors and may provide limited guidance for intervention efforts. Therefore, alternative methods (i.e., SEM) were used to examine the psychosocial burden formed by the interaction of psychosocial factors on ART adherence. The second-order SEM had a better goodness of fit and explained more variation in ART adherence than the first-order SEM. The second-order SEM showed differential effects of multiple psychosocial factors, supporting the syndemic theory that multiple psychosocial factors coexisted and interacted to influence the health outcomes [13]. Medication beliefs and self-efficacy explained the most variation of the psychosocial effect positively associated with ART adherence, followed by supportive environment and negative emotions. These findings highlight that the medication beliefs and self-efficacy may be a priority for improvement in resource-limited settings.

Several limitations should be noted. First, due to the nature of the cross-sectional design, causality cannot be inferred and prospective cohort studies are needed. Second, convenience sampling was used to recruit PLHIV due to the lack of sampling frameworks for hard-to-reach populations [24]. However, six cities were selected based on geographic location and economic development, which may alleviate the concerns about representativeness and extrapolation. Third, participants included in the current study were followed up one year after the baseline survey, their ART adherence may be overestimated because some participants at baseline were lost to follow-up, who may be poorly adherent to ART [63]. Finally, self-reported ART adherence often overestimates actual treatment adherence [64]. A previous study showed a high proportion of PLHIV in Yunnan Province reporting optimal adherence, compared with much lower optimal adherence using objective methods (pill count, electronic drug monitor) and with no association with viral load [43]. However, it is worth noting that a recent review concluded that no measure of ART adherence consistently provided either sufficiently sufficient sensitivity or specificity to detect viral non-suppression [1]. Nevertheless, objective measures such as plasma drug concentrations or dried blood spots were warranted to better measure ART adherence [65].

## Conclusion

In summary, about nine out of ten PLHIV on ART in Guangdong Province self-reported optimal ART adherence. However, lack of medication reminders, side effects of ART affecting daily life, and unemployment remained barriers to optimal ART adherence. More efforts should be made to provide patient-centered care to reduce the side effects of ART and to find appropriate treatment options that fit into the lives of PLHIV. In addition, comprehensive interventions to enhance the medication beliefs and self-efficacy, provide a supportive environment and reduce negative emotions may be helpful in improving ART adherence. In particular, strengthening the medication beliefs and self-efficacy may be a priority in resource-limited settings.

### Electronic supplementary material

Below is the link to the electronic supplementary material.

Supplementary Material 1

## Data Availability

The datasets used and/or analyzed during the current study are available from the corresponding authors on reasonable request.
