# Supplementary material for: Factors associated with antiretroviral treatment adherence among people living with HIV in Guangdong Province, China: a cross sectional analysis
Source: BMC Public Health. 2024 May 20;24:1358. doi: 10.1186/s12889-024-18774-6 (PMC11106959; doi:10.1186/s12889-024-18774-6)
Supplement: Supplementary file 1 — Supplementary Material 1 [file 12889_2024_18774_MOESM1_ESM.docx]

Supplementary Table 1. The model fit of the first-order structural equation model

|  | Index | Criterion | model |
| --- | --- | --- | --- |
| Absolute fit index | *χ*^2^/*df* | <3.00 | 5.59 |
|  | GFI | >0.90 | 0.93 |
|  | RMR | <0.05 | 0.07 |
| Parsimony fit index | RMSEA | ≤0.08 | 0.07 |
|  | PGFI | >0.50 | 0.75 |
|  | AGFI | >0.90 | 0.90 |
| Comparative fit index | NFI | >0.80 | 0.82 |
|  | CFI | >0.80 | 0.85 |

df: degrees of freedom; GFI: goodness-of-fit index; RMR: root mean square residual; RMESA: the root mean square error of approximation; PGFI : parsimonious goodness-of-fit index; AGFI: adjusted goodness-of-fit index; NFI: normed fit index CFI: comparative fit index.

Supplementary Table 2. The estimating parameters of the first-order structural equation model

| Path | standardized coefficient | standard error | *t* | *P* | *R^2^* |
| --- | --- | --- | --- | --- | --- |
| The beliefs about medicines ← Psychosocial factor | 0.300 | 0.044 | 6.824 | **<0.001** | 0.090 |
| Adherence self-efficacy ← Psychosocial factor | 0.357 | 0.043 | 8.299 | **<0.001** | 0.127 |
| Depressive symptoms ← Psychosocial factor | -0.336 | 0.043 | -7.737 | **<0.001** | 0.113 |
| HIV-related stigma ← Psychosocial factor | -0.134 | 0.046 | -2.912 | **0.004** | 0.018 |
| Social cohesion ← Psychosocial factor | 0.575 | 0.039 | 14.572 | **<0.001** | 0.331 |
| Social participation ← Psychosocial factor | 0.157 | 0.046 | 3.418 | **<0.001** | 0.025 |
| Collective engagement ← Psychosocial factor | 0.317 | 0.044 | 7.245 | **<0.001** | 0.100 |
| Social trust ← Psychosocial factor | 0.430 | 0.042 | 10.324 | **<0.001** | 0.185 |
| Social support ← Psychosocial factor | 0.536 | 0.040 | 13.424 | **<0.001** | 0.287 |
| Frequency of taking medications on time ← ART adherence factor | 0.646 | 0.024 | 26.805 | **<0.001** | 0.417 |
| Average number of days per week missed with at least one missed dose ← ART adherence factor | 0.896 | 0.016 | 54.673 | **<0.001** | 0.802 |
| Last time at least one dose of ART medication was missed ← ART adherence factor | 0.880 | 0.017 | 52.836 | **<0.001** | 0.774 |
| ART adherence factor ← Psychosocial factor | 0.249 | 0.047 | 5.234 | **<0.001** | 0.062 |

Supplementary Table 3. The model fit of the second-order structural equation model

|  | Index | Criterion | model |
| --- | --- | --- | --- |
| Absolute fit index | *χ*^2^/*df* | <3.00 | 2.98 |
|  | GFI | >0.90 | 0.97 |
|  | RMR | <0.05 | 0.05 |
| Parsimony fit index | RMSEA | ≤0.08 | 0.05 |
|  | PGFI | >0.50 | 0.73 |
|  | AGFI | >0.90 | 0.95 |
| Comparative fit index | NFI | >0.80 | 0.91 |
|  | CFI | >0.80 | 0.94 |

df: degrees of freedom; GFI: goodness-of-fit index; RMR: root mean square residual; RMESA: the root mean square error of approximation; PGFI : parsimonious goodness-of-fit index; AGFI: adjusted goodness-of-fit index; NFI: normed fit index CFI: comparative fit index.

Supplementary Table 4. The estimating parameters of the second-order structural equation model

| Path | standardized coefficient | standard error | *t* | *P* | *R^2^* |
| --- | --- | --- | --- | --- | --- |
| The beliefs about medicines ← Medication beliefs and self-efficacy factor | 0.456 | 0.053 | 8.557 | **<0.001** | 0.208 |
| Adherence self-efficacy← Medication beliefs and self-efficacy factor | 0.759 | 0.076 | 10.012 | **<0.001** | 0.576 |
| Depression ← Negative emotions factor | 1.054 | 0.094 | 3.591 | **<0.001** | / |
| HIV-related stigma ←Negative emotions factor | 0.253 | 0.079 | 3.224 | **0.001** | 0.064 |
| Social cohesion ← Supportive environment factor | 0.622 | 0.042 | 14.868 | **<0.001** | 0.386 |
| Social participation ← Supportive environment factor | 0.172 | 0.046 | 3.729 | **<0.001** | 0.030 |
| Collective engagement ← Supportive environment factor | 0.370 | 0.043 | 8.512 | **<0.001** | 0.137 |
| Social trust ← Supportive environment factor | 0.442 | 0.042 | 10.444 | **<0.001** | 0.195 |
| Social support ← Supportive environment factor | 0.557 | 0.042 | 13.417 | **<0.001** | 0.310 |
| Frequency of taking medications on time ← ART adherence factor | 0.647 | 0.024 | 26.954 | **<0.001** | 0.419 |
| Average number of days per week missed with at least one missed dose ←ART adherence factor | 0.894 | 0.016 | 55.723 | **<0.001** | 0.800 |
| Last time at least one dose of ART medication was missed ← ART adherence factor | 0.880 | 0.016 | 53.987 | **<0.001** | 0.775 |
| Medication beliefs and self-efficacy factor ← Psychosocial factor | 0.650 | 0.085 | 7.635 | **<0.001** | 0.423 |
| Negative emotions factor ← Psychosocial factor | -0.376 | 0.118 | -3.191 | **0.001** | 0.141 |
| Supportive environment factor ← Psychosocial factor | 0.503 | 0.069 | 7.340 | **<0.001** | 0.253 |
| ART adherence factor ← Psychosocial factor | 0.431 | 0.057 | 7.561 | **<0.001** | 0.186 |
